# DLEC1 Expression Is Modulated by Epigenetic Modifications in Hepatocelluar Carcinoma Cells: Role of HBx Genotypes

**DOI:** 10.3390/cancers2031689

**Published:** 2010-09-16

**Authors:** Dandan Niu, Huixing Feng, Wei Ning Chen

**Affiliations:** School of Chemical and Biomedical Engineering, Nanyang Technological University, Singapore; E-Mails: niud0001@e.ntu.edu.sg (D.N.); HXFeng@ntu.edu.sg (H.F.)

**Keywords:** DLEC1, HBV, HBx, DNA methylation, histone acetylation

## Abstract

Deleted in Lung and Esophageal Cancer 1 (DLEC1) is a functional tumor suppressor gene (TSG). It has been found to be silenced in a variety of human cancers including hepatocellular carcinoma (HCC). The silencing of DLEC1 can be modulated by epigenetic modifications, such as DNA hypermethylation and histone hypoacetylation. In the case of HCC, hepatitis B virus X protein (HBx) has been implicated in methylation of target promoters resulting in the down-regulation of tumor suppressor genes, which in turn contributes to the development of HCC. In the present study, we first established a cell system in which epigenetic modifications can be modulated using inhibitors of either DNA methylation or histone deacetylation. The cell system was used to reveal that the expression of DLEC1 was upregulated by HBx in a genotype-dependent manner. In particular, HBx genotype A was found to decrease DNA methylation of the DLEC1 promoter. Our results have provided new insights on the impact of HBx in HCC development by epigenetic modifications.

## 1. Introduction

Numerous genetic abnormalities have been implicated in the development of a variety of human cancers including hepatocellular carcinoma (HCC). These include chromosomal deletions as well as mutations in tumor suppressor genes (TSGs) [1,2]. Recently, epigenetic modifications have been suggested in HCC development [3], involving both DNA methylation and histone acetylation status. DNA methylation of promoter regions, especially the CpG islands, has been shown to modulate chromatin stability and in turn impacts on the expression of target genes [3]. DNA hypermethylation has been linked to the down-regulation of several TSGs including RASSF1A, p16^INK14^, E-cadherin [3,4], GSTP1 [5] and DLEC1 [6]. DNA methylation is catalyzed by DNA methyltransferases (DNMTs), with DNMT1 as the most active one [7,8].

On the other hand, histone acetylation affects the lysine residues at the N terminus of histone proteins resulting in the removal of positive charges, thus reducing the affinity between histones and DNA [7]. The modification allows RNA polymerase and transcription factors greater access to the promoter region [9]. As a result, histone acetylation enhances transcription while histone deacetylation represses transcription. Histone acetylation is catalyzed by histone acetyltransferases (HATs) and histone deacetylation is catalyzed by histone deacetylases (HDACs) [11,12].

Deleted in Lung and Esophageal Cancer 1 (DLEC1) is a functional tumor suppressor gene (TSG). It has been found to be silenced in a variety of human cancers including non-small cell lung cancer [10], esophageal cancer [11], colon and gastric cancers [12,13], nasopharyngeal cancer [14], epithelial ovarian cancer, and HCC [6]. While other TSGs have been reported to be modulated exclusively by DNA methylation [3,14,15], the silencing of DLEC1 involves both DNA methylation and histone acetylation [11,16].

HBV has been implicated in epigenetic modifications leading to HCC. In particular, the versatile smallest viral protein HBV X protein (HBx) [17] has been involved in DNA methylation of target promoters including TSGs, resulting in their down-regulation and eventually the development of HCC [3]. It has been suggested that HBx modulates the process of DNA methylation by regulating the expression of DNMT 1 [3,18]. For example, the expression of E-cadherin [3,4] and p16^INK14^ [18] can be repressed upon the activation of DNMT1 by HBx. Similarly, HBx has been shown to induce the expression of MTA1 and HDAC1, thus activating the hypoxia signaling in HCC cells [19].

Recent evidence has linked HBV genotypes to distinct clinic manifestations. For example, genotype B appears to be closely associated with HCC whereas genotype A and C are often found in hepatitis and cirrhosis samples [20]. We have recently reported the genotype-related effects on cellular apoptosis [18]. Further molecular characterization of HBV genotypes in epigenetic modifications should provide us with helpful information on the underlying mechanisms of genotype-specific HCC development. 

In the present report, the effect of HBx on regulating DLEC1 through epigenetic mechanisms (including DNA methylation and histone acetylation) was analyzed. HBx genotype A was found to decrease DNA methylation while increasing the level of histone acetylation, leading to the up-regulation of DLEC1. Our results provide new insights on the impact of HBx in HCC development by epigenetic modifications.

## 2. Results and Discussions

### 2.1. DLEC1 Expression Is Altered by Epigenetic Regulations in HCC Cell Lines

DLEC1 has been found to be silenced in human cancers including HCC. Consistently, its overexpression in cell lines led to a decrease in colony formation, cell growth and cell size, and an induction of G1 arrest in the cell cycle [6]. The silencing of DLEC1 is associated with DNA methylation [6,10], as well as histone acetylation [11,16,21]. However, the reported epigenetic studies of DLEC1 have been focused on clinical observations in human cancers without revealing underlying mechanisms. 

In the case of HCC associated with HBV infection, epigenetic modifications may well implicate viral proteins including HBx, which has been linked to the silencing of tumor suppressor genes and development of HCC [3]. Therefore in this study, we focused on DLEC1 expression in HCC cell lines, and explored the role of HBV replication and HBx protein in the regulation of DLEC1 expression.

To assess the status of DNA methylation in the DLEC1 promoter, we performed bisulfite genome sequencing (BGS) of the DLEC1 promoter in two of HCC cell lines, HepG2 and Huh7. 

**Figure 1:**
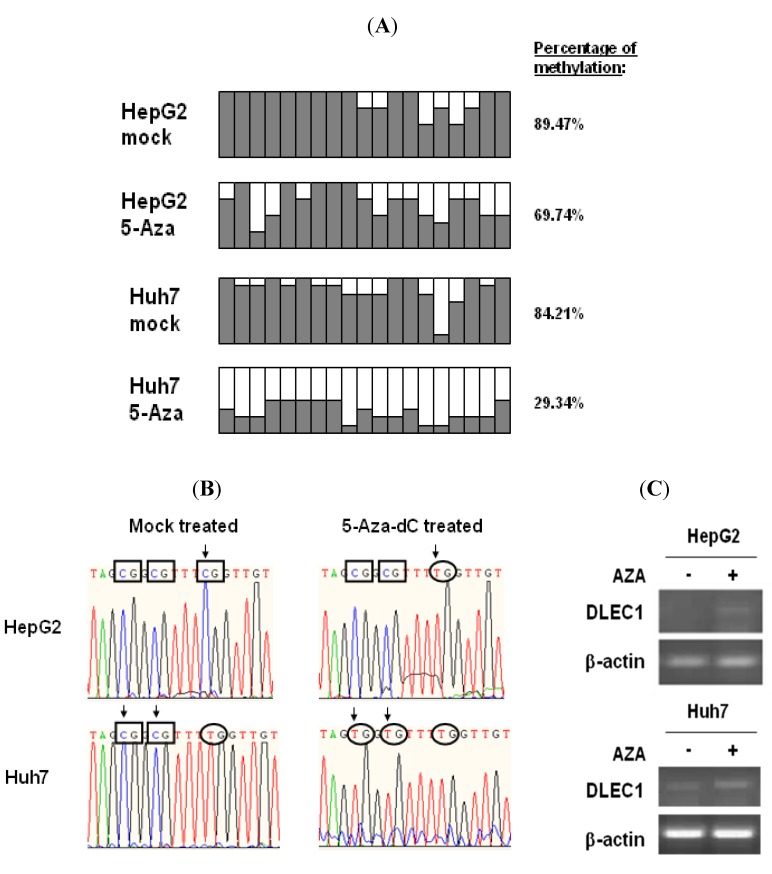
DLEC1 promoter hypermethylation and its expression in HCC cell lines. (**A**) Promoter methylation analysis by bisulfite genome sequencing of DLEC1 5’ CpG sites in both of HepG2 and Huh7 with or without treatment of 5-Aza-dC. 19 CpG dinucleotides were analyzed in the 5’ promoter of DLEC1 (represented as 19 columns). 6–8 clones of each sample were sequenced and the results were presented as follow: Open area indicates the percentage of unmethylated CpG sites, while dark area indicates percentage of methylated CpG sites. The quantitative result in percentage of methylated CpG sites was listed on the right of the column groups. Three independent experiments were carried out and representative result from one of them is listed here. (**B**) Representative bisulfite sequencing results of the DLEC1 promoter region showing the CpG methylation status of bisulfite treated DNA from two cell lines mock treated or 5-Aza-dC treated. Rectangles indicate the methylated CpG sites, while ellipses for unmethylated CpG sites. Arrows mark the CpG sites whose methylation status was changed by 5-Aza-dC treatment. (**C**) RT-PCR analysis of DLEC1 expression in two cell lines mock treated or 5-Aza-dC treated.

The DLEC1 promoters of the above cell lines were analyzed after the treatment of DNMT inhibitor 5-Aza-dC or mock treatment. Results shown in Figure 1A indicate that the DLEC1 promoter was highly methylated in both Huh7 (84.21%, Figure 1A row 1) and HepG2 cells (89.47%, Figure 1A row 3). On the other hand, methylation in the DLEC1 promoter was decreased after the cells were incubated with the DNMT inhibitor 5-Aza-dC. Specifically, the methylation was found to be at 29.34% in Huh7 (Figure 1A row 2), 69.74% in HepG2 (Figure 1A row 4). Our findings in HepG2 were consistent with the reported investigation on the methylation of the DLEC1 promoter in HepG2 [6]. Combined with our findings in Huh7 cells, our results indicated that DLEC1 was significantly methylated in HCC-derived cell lines.

Representative BGS results of CpG methylation status changed before and after incubation of the cells with 5-Aza-dC are shown in Figure 1B. In HepG2 cells, the three presented CpG sites in DLEC1 promoter were methylated before incubation with 5-Aza-dC (Figure 1B, upper left panel); while one of them (rectangle in left panel with arrow) was demethylated after incubation (Figure 1B, upper right panel). The methylation status of the same three CpG sites was different in Huh7 cells. Before the incubation of the cells with 5-Aza-dC, two of the CpG sites were methylated (lower left panel, Figure 1B), whereas these two CpG sites were demethylated after incubation (lower right panel, Figure 1B). 

To correlate the methylation of DLEC1 promoter with its expression, RT-PCR was performed in both HepG2 and Huh7 cells. As shown in Figure 1C, DLEC1 expression was upregulated in both types of cells incubated with 5-Aza-dC compared with those without any incubation with 5-Aza-dC. Our results therefore suggest that DLEC1 expression was inhibited in HepG2 and Huh7 cells and the DNA methylation was directly involved in this suppression. 

**Figure 2:**
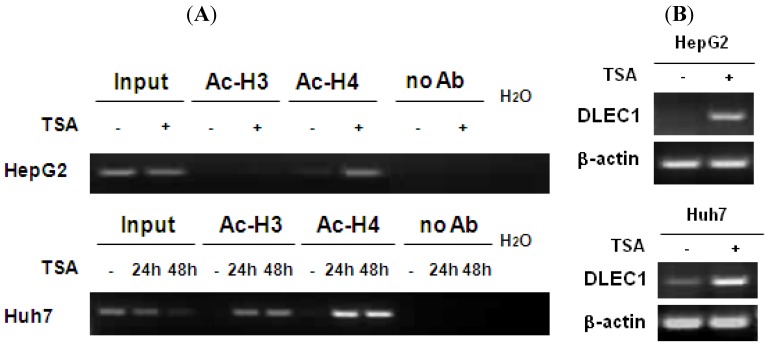
Histone H3 and H4 hypoacetylation in DLEC1 5’ region and its expression in HCC cell lines. (**A**) ChIP analysis of the 5’ region of DLEC1 in HepG2 and Huh7 mock treated or TSA treated. DNA sequences cross-linked to acetylated histone H3 and H4 were co-immunoprecipitated as part of the chromatin complex with antibodies specific to acetylated histone H3 and H4. The reaction without antibody immunoprecipitation (no Ab) serves as negative control. Input indicates 1% of total DNA prior to immunoprecipitation. (**B**) RT-PCR analysis of DLEC1 expression in two cell lines mock treated or TSA treated.

In addition to DNA methylation, the involvement of histone acetylation in regulating DLEC1 expression was analyzed by chromatin immunoprecipitation (ChIP) assay. The results shown in Figure 2A indicate that low level of histone H4 acetylation was detected in both HepG2 (lane 5, upper panel) and Huh7 (lane 7, lower panel). In addition, the acetylation of histone H3 was not detected in HepG2 (lane 3, upper panel) and Huh7 (lane 4, lower panel). The acetylation levels for both histones 3 and 4 increased in Huh7 cells (lanes 5, 6, 8 and 9, lower panel) incubated with the histone deacetylase inhibitor TSA, for HepG2, the acetylated histone H4 was significantly increased (lanes 6, upper panel), while the increase of acetylation of histone H3 was less significant (lanes 4, upper panel). 

To correlate the histone acetylation with DLEC1 expression, RT-PCR was performed. Results shown in Figure 2B indicate that the expression of DLEC1 was reactivated in both HepG2 cells (upper panel) and Huh7 cells (lower panel) after their incubation with TSA. Hence, DLEC1 expression was significantly correlated with their histone acetylation status (Figure 2B). Interestingly, the increase of DLEC1 expression after the incubation of cells with TSA (Figure 2B) was more significant than that in cells incubated with 5-Aza-dC (Figure 1C). These results suggest that both DNA hypermethylation and histone hypoacetylation are involved in inhibiting DLEC1 expression, with histone acetylation having a more active role. 

### 2.2. DLEC1 Is Inhibited in HBV-Producing HepG2.2.15 Cells

**Figure 3:**
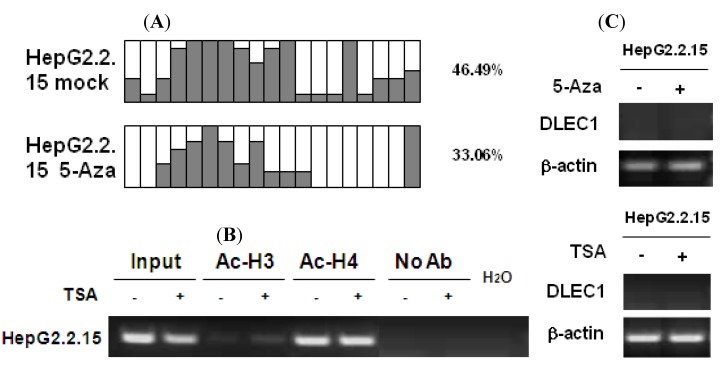
DNA methylation and histone acetylation status of DLEC1 and its expression in HepG2.2.15 cells. (**A**) Promoter methylation analysis by bisulfite genome sequencing of DLEC1 5’ CpG sites in HepG2.2.15 with or without treatment of 5-Aza-dC. Open area indicates the percentage of unmethylated CpG sites, while dark area indicates percentage of methylated CpG sites. The quantitative result in percentage of methylated CpG sites is listed on the right of the column groups. Three independent experiments were carried out and representative result from one of them was listed here. (**B**) ChIP analysis of 5’ region of DLEC1 in HepG2.2.15 mock treated or TSA treated. Input indicated 1% of total DNA prior to immunoprecipitation. (**C**) RT-PCR analysis of DLEC1 expression in HepG2.2.15 treated by either 5-Aza-dC or TSA.

To investigate the effects of HBV replication on epigenetic modifications in the context of DLEC1 expression, analyses on DNA methylation and histone acetylation were carried out in HepG2.2.15 cells which constitutively produce HBV particles. HepG2.2.15 is derived from HepG2 cells with integration of a replicative HBV genome (genotype D), and it supports both assembly and secretion of replicative intermediates of HBV DNA and Dane particles [22]. Results of methylation analysis by BGS shown in Figure 3A indicated that the methylation of DLEC1 promoter was lower in HepG2.2.15 (46.49%) than that in HepG2 (89.47%, Figure 1A). It was further decreased to 33.06% after the incubation of the cells with 5-Aza-dC (Figure 3A). In addition, the acetylation level of histones H3 and H4 was found to be higher in HepG2.2.15 (lanes 3 and 5, Figure 3B) compared with that in HepG2 (lanes 3 and 5, Figure 2A). The level of acetylation of histone 3 was further increased after HepG2.2.15 cells were incubated with TSA (lane 4, Figure 3B). 

In contrast to HepG2 and Huh7 cells, the expression of DLEC1 in HepG2.2.15 was not detectable by RT-PCR (Figure 3C). DLEC1 expression was not reactivated in HepG2.2.15 cells incubated with either 5-Aza-dC or TSA (Figure 3C)

Our results therefore suggest that HBV replication is involved in the suppression of DLEC1 in HepG2.2.15, and likely to be independent of the epigenetic mechanisms (including both DNA methylation and histone acetylation). However, the exact underlying mechanism remains unclear. On the other hand, since the HBV genotype expressed in HepG2.2.15 was genotype D, the association between HBV genotype and DLEC1 regulation was further investigated. 

### 2.3. DLEC1 Expression Is Modulated by HBx Genotypes

**Figure 4:**
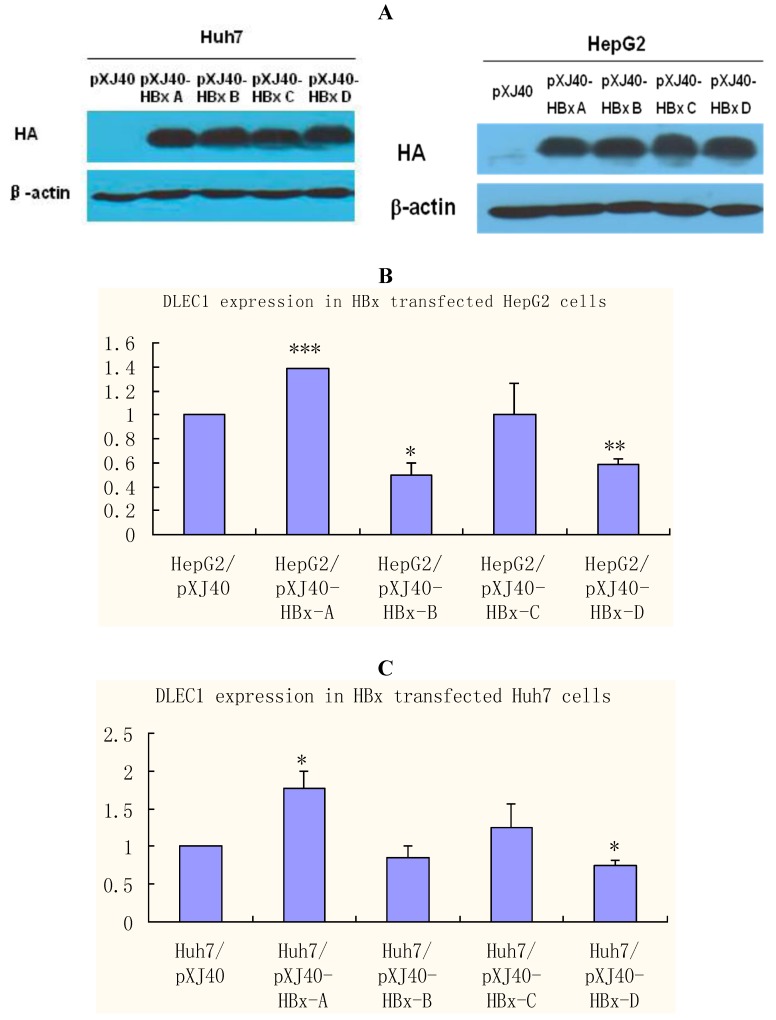
DLEC1 expression in HBx genotype A-D expressing HepG2 and Huh7. (**A**) Western analysis of HBx expression in HepG2 and Huh7 cells. HBx expression was detected by HA tag contained in the vector pXJ40. β-actin was detected as the internal control. (**B**) Analysis of DLEC1 expression in HBx genotype A-D expressing HepG2 by real-time RT-PCR. (**C**) Analysis of DLEC1 expression in HBx genotype A-D expressing Huh7 by real-time RT-PCR. Experiment was done three times. pXJ40 was included as an empty vector control. Experimental differences were tested for statistical significance using t-test. * p < 0.05; ** p < 0.01; ***p < 0.001.

Among the HBV proteins, HBx has been suggested in epigenetic regulation of gene expression [3,18] and recent evidence has revealed genotype specific effects of HBx in host cells [15,23]. HBx was therefore selected to investigate further the role of HBV in epigenetic regulations of DLEC1. HepG2 and Huh7 cells were transfected separately with plasmids containing HBx of different genotypes (A, B, C and D). The results of Western blot analysis shown in Figure 4A indicate that the expression of HBx was comparable among different genotypes, whereas no HBx was detected in control cells transfected with the empty plasmid pXJ40. 

DLEC1 expression was then examined in cells transfected with HBx of various genotypes by real-time RT-PCR (Figure 4B and C). The results in HepG2 cells shown in Figure 4B indicate that DLEC1 expression was modulated in relation to HBx genotypes. In comparison with the level in cells transfected with the empty plasmid pXJ40 (column 1), DLEC1 expression was significantly increased in cells transfected with HBx genotype A (column 2), and significantly decreased in cells transfected with either HBx genotype B or D (columns 3 and 5, respectively). In contrast, there was no significant effect of HBx C on expression of DLEC1 compared with that in cells transfected with pXJ40 (column 4). A similar pattern was observed in Huh7 cells (Figure 4C). 

Taken together, our results suggest that DLEC1 expression is modulated differently in cells transfected with different HBx genotypes.

The biological significance of demethylation/acetylation of DLEC1 and its up-regulation HBx may be of particular interest. HBx has been generally considered as an onco-protein that promotes tumorigenesis. Our results appeared to suggest the opposite, in that HBx enhanced the expression of the tumor suppressor gene, DLEC1 leading to the suppression of tumor formation.

HBx has been shown to be a multifunctional viral protein involved in a number of cellular activities with contrasting effects. Therefore, it is not unexpected to see some contradictory phenotypes related to HBx functions, such as the role of HBx in apoptosis [24], both pro-apoptotic [15,25] and anti-apoptotic [26,27]. Our findings on the upregulation of TSGs by HBx were consistent with reports on other TSGs. For example, for SOCS-1, which is silenced by hypermethylation in HCC and shows growth-suppression activity [28], its CpG island hypermethylation has been found to be inversely associated with HBV infection status [29]. Similarly, IGFBP3, a potential tumor suppressor previously reported to be repressed in HCC, has been found to be both downregulated [3] and upregulated [30] by HBx. This contradiction suggests a role of HBx genotypes.

Therefore, the effects of HBx genotypes on the regulation of DLEC1 expression as well as epigenetic alterations of DLEC1 promoter were further investigated in this study.

### 2.4. DLEC1 Is Modulated by HBx-Modulated Epigenetic Modifications

**Figure 5:**
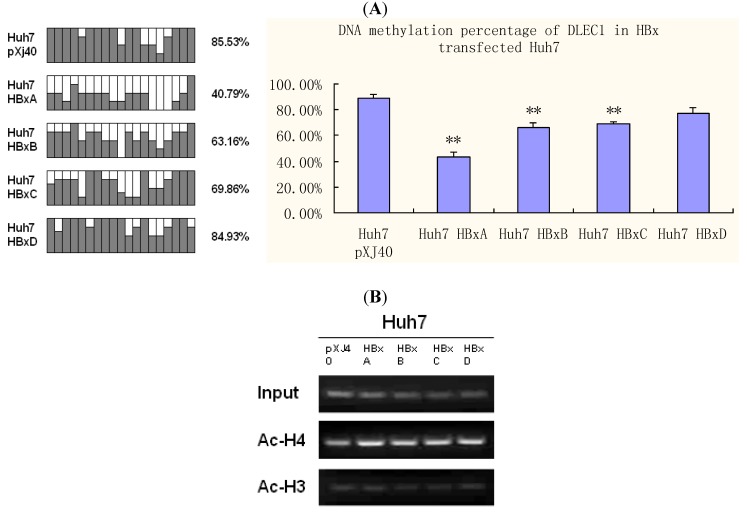
DNA methylation and histone acetylation status of DLEC1 in Huh7 expressing HBx genotypes A-D. (**A**) Promoter methylation analysis by bisulfite genome sequencing of DLEC1 5’ CpG sites in Huh7 transfected by HBx genotypes A-D. Open area indicates the percentage of unmethylated CpG sites, while dark area indicates percentage of methylated CpG sites. The quantitative result in percentage of methylated CpG sites is listed on the right of the column groups. Three independent experiments were carried out and representative result from one of them was listed here as the upper panel; the lower panel summarized the results from all three experiments. (**B**) ChIP analysis of the 5’ region of DLEC1 in Huh7 expressing HBx. Input indicates 1% of total DNA prior to immunoprecipitation. pXJ40 was included as an empty vector control. Experiments were done three times. Experimental differences were tested for statistical significance using t-test. * p < 0.05; **p < 0.01.

Inspired by the genotype specific effects of HBx on DLEC1 expression, correlation of such effects to epigenetic alterations was next examined. Both BGS and ChIP assay were performed to evaluate the DNA methylation and histone acetylation status in Huh7 cells transfected with HBx of different genotypes (Figure 5).

The results shown in Figure 5A indicate that DNA methylation of the DLEC1 promoter in Huh7 transfected by HBx A-D varied from each other. The lowest percentage of methylation was in Huh7 transfected by HBx A (40.79%), which was half of that observed in cells transfected by vector pXJ40 (85.53%). HBx genotype B (63.16%) and C (69.86%) also led to lower percentage of methylation than vector, but HBx D (84.93%) had no obvious effect on DNA methylation compared with the vector control.

On the other hand, histone acetylation status was explored by ChIP as indicated in Figure 5B. One percent of the total DNA before the immunoprecipitation was used as the input control. Compared with the input control, the acetylated histone H4 (Figure 5B, panel 2) was increased to a different extent in Huh7 transfected by HBx of various genotypes, especially in HBx A (Figure 5B, panel 2, lane 2), compared with that in cells transfected with pXJ40 (lane 1). No significant changes in the acetylation of histone 3 were observed in cells transfected with pXJ40 or individual HBx genotypes (Figure 5B, panel 3).

Combined with DNA methylation alterations, our results suggest a significant role of HBx genotype A in inducing epigenetic modifications, which in turn modulates the expression of DLEC1. Our results therefore indicate that the DLEC1 expression is modulated by epigenetic modifications, which are influenced by HBx in a genotype specific manner.

Among the four genotypes of HBx, HBx A showed significant correlation between epigenetic analyses and DLEC1 expression. Interestingly, HBx D had no significant effect on DNA methylation (Figure 5A) nor on the histone acetylation. However, its expression led to a decrease in DLEC1 expression. Our findings indicate that the HBV genotype D repressed DLEC1 in HepG2.2.15 independently of the epigenetic alterations in its promoter (part 2.2). However, the underlying mechanism of repression of DLEC1 by HBV/HBx genotype D remains to be explored. 

The mechanism of genotype-specific effects of HBx on epigenetic alterations of DLEC1 needs further characterization. One possible focus for such investigations may be the proline-rich region identified in our lab [23]. This proline-rich region is hitherto the only noticeable variable region among various HBx genotypes. In addition to its role in interacting with cytoskeletal proteins [31,32], further investigation should shed new light on the role of this proline-rich region in HBx-mediated epigenetic alterations. Another possible mechanism could be involved here is the direct interaction of HBx with *de novo* DNA methyltransferase DNMT3A [30]. It has been reported that HBx recruits DNMT3A to the promoter of some genes and subsequently silenced their transcription via de novo DNA methylation; HBx can also trigger the transcription of some genes via deprivation of DNMT3A from their promoters [30]. In our case, transcription of DLEC1 could be affected by HBx A via deprivation of DNMT3A from its promoter and thus upregulated. The sequence variations among different genotypes of HBx may as well affect the interaction of HBx with DNMT3A and lead to genotype specific effects on DLEC1 expression. The detailed mechanism remains to be explored.

## 3. Experimental Section

### 3.1. Plasmid Construction

HBV genomes of various genotypes were all from Singapore clinical samples, and were cloned as replicative genomes as previously described [31,33]. HBx coding sequences of distinct genotypes (A–D) were amplified from the respective HBV genomes by PCR and inserted into HindIII/ PstI site of the vector plasmid pXJ40 with higher expression efficiency and a HA tag [15,32], which was a gift from Dr. Cheng Gee Koh (SBS, NTU). All the primers used were as those in our previous report [15], and the resulted constructs were confirmed by sequencing.

### 3.2. Cell Culture and Transfection

HepG2 and Huh7 cell lines were maintained in minimal essential medium (MEM) supplemented with 10% fetal bovine serum (FBS) (Gibco, Invitrogen) in an incubator containing 5% CO_2_ at 37 °C. For transfection, electro-transfection from Amaxa system was used as up to 50% for HepG2 and 85% for Huh7 of the efficiency was achieved (data not shown) using Cell Line Nucleofector Kit V (Amaxa Inc, USA). The transfection program used for both of the cell lines was T-028 provided in the Amaxa system. HepG2.2.15 cells were maintained with Dulbecco's modified eagle's medium (DMEM) supplemented with 10% FBS, 1 × nonessential amino acid (Gibco, Invitrogen), and 150 μg/mL of G418 (Geneticin) (Gibco, Invitrogen), in an incubator containing 5% CO_2_ at 37 °C. 

### 3.3. 5-Aza-2’-Deoxycytidine (5-Aza-dC) and Trichostatin A (TSA) Treatment of Cells

For the 5-Aza-dC treatment, cells were seeded in low density 12–18 hours before the treatment. Cells were treated from a DMSO (Sigma) dissolved stock solution (10 mM) of 5-Aza-dC (A3656, Sigma-Aldrich) at a final concentration of 5 μM, or were mock-treated by adding into the medium with the same volume of DMSO alone. Treatment was conducted for 96 hours, with the drug and culture medium replaced every 24 hours [5,34]. For the TSA treatment, the cells were treated from a DMSO dissolved stock solution (5 mM) of TSA (T8552, Sigma-Aldrich) at a final concentration of 500 ng/mL [11,16], or were mock-treated by adding into the medium with the same volume of DMSO only. Treatment was conducted for 24-48 hours, with the drug and culture medium replaced every 24 hours.

### 3.4. RNA Extraction and Quantification

Total RNA of the cells transfected with pXJ40 vector or plasmids containing HBx or treated by 5-Aza-dC or TSA was isolated using RNeasy Mini kit (QIAGEN). Briefly, buffer RTL was added to cell pellet, followed by addition of 1 volume 70% ethanol. The mixture was transferred to RNeasy mini column and pelleted in a bench top centrifuge (Sigma). The flow-through was discarded and the column washed with buffer RW1 and buffer RPE, respectively. RNase-free water was added directly onto the membrane of the column and RNA was eluted by centrifugation. The concentration of RNA was determined by measuring the absorbance at 260 nm (A_260_) in a spectrophotometer, and the quality was examined by the ratio of absorbance at 260 nm to 280 nm (A_260_/A_280_). 

### 3.5. RT-PCR and Real-Time RT-PCR

RT-PCR was performed with the help of OneStep RT-PCR kit (QIAGEN), according to the manufacturer’s instructions. The RT-PCR program was 50 °C 30 min for reverse transcription; 95 °C 15 min for initial PCR activation; then 35 cycles of 40 s 94 °C denaturation, 40 s 55 °C annealing and 1 min 72 °C for extension; followed by a final extension of 72 °C 10 mins. Primers targeting DLEC1 were DLEC1RTf/r as listed in Table 1. For the internal control, we used primers targeting β-actin: β-actinRTf/r (Table 1).

**Table 1:** Primers used for RT-PCR, BGS and ChIP.

Primer name	Sequence	Product size	Annealing temperature	Reference
DLEC1RTf	5’-TTCCTCCCTCGCCTACTC-3’	310 bp	55 °C	[10]
DLEC1RTr	5’-AAACTCATCCAGCCGCTG-3’			
β-actinRTf	5’-CTTAGTTGCGTTACACCCTTTC-3’	151 bp	55 °C	-
β-actinRTr	5’-ACCTTCACCGTTCCAGTTTT-3’			
DLEC1BGS1	5’-GAAGATATAAATGTTTATAATGATT-3’	291 bp	50 °C	[6]
DLEC1BGS8	5’-ACTATAATAAAAAACCTCAAAATAAA-3’			
DLEC1ChIPf	5’-ACAATGACCACAGCGATGAC-3’	275 bp	65 °C	[16]
DLEC1ChIPr	5’-GCTGTAGTGGAAGGCCTCAG-3’			

Real-time RT-PCR was performed by using iScript One-step RT-PCR kit (Bio-Rad) according to the instructions, to accurately quantify the original amount of target mRNAs in different samples. The real-time RT-PCR was carried out in an IQ5 multicolor real-time PCR detection system (Bio-Rad), with the cycling program: 50 °C 10 mins for cDNA synthesis; 95 °C 5 mins for iScript Reverse Transcriptase inactivation; then 45 cycles of PCR and detection as the following: 95 °C 30 s, 55 °C 30 s, 72 °C 30 s; and a melt curve analysis of 95 °C 1 min, 55 °C 1 min, 55 °C 10 s with 80 cycles increasing each by 0.5 °C each cycle. 

Microsoft Excel formatted data, which could be automatically provided by the IQ5 optical system software version 2.0 (Bio-Rad) included amplification analysis, experimental report, melt curve analysis and threshold cycle number. The fold changes were calculated using the following formula:

Sample ΔCt = Ct _sample_ − Ct _β-actin_

ΔΔCt = Sample ΔCt − control ΔCt

the fold of sample *vs.* control = 2^ΔΔCt^.

### 3.6. Western Blotting

Cell extracts were prepared in a home-made TNT lysis buffer (50 mM Tris-HCl, pH 7.4, 150 mM NaCl, 1% Triton-100, and a protease inhibitor cocktail), followed by protein concentration quantification with Bradford Dye Reagent (#500-0205, Bio-Rad). After that, equal amounts (30 μg) of cell lysates were resolved by 12% SDS-PAGE. The resolved proteins were electro-transferred to PVDF membranes (Bio-Rad), which were then probed with a primary antibody: either anti-HA (F-7: sc-7392, Santa Cruz Biotechnology) at 1:1000 dilution, or anti-β-actin (AC-74, Sigma) at 1:10,000 dilution. A horseradish peroxidase (HRP)-conjugated anti-mouse IgG antibody (#31430, PIERCE) was used as the secondary antibody with a 1:5000 dilution. The results were visualized using Supersignal West solutions (#1856136, PIERCE).

### 3.7. Genomic DNA Extraction and Quantification

Genomic DNA of cells was extracted with PureLink Genomic DNA Kit (Invitrogen), according to the user manual provided with the kit. Briefly, genomic DNA was isolated by digestion of protein and RNA with Proteinase K and RNase A in the Lysis/Binding Buffer during an incubation of 10 mins at 55 °C. After that, ethanol was added and samples were applied to the PureLink Spin Column. Centrifugation was performed to allow the DNA to bind to the column, followed by washing and eluting. The purity of the DNA extract was evaluated by spectrophotometer determination of the A_260_/A_280_ ratio, while the concentration was calculated according to the A_260_.

### 3.8. Bisulfite Conversion and Bisulfite Genome Sequencing (BGS)

Genomic DNA was treated using EpiTech Bisulfite Kit (QIAGEN) for complete bisulfite conversion and cleanup for the following methylation analysis. The bisulfite conversion was conducted following the manufacturer’s instructions. 1.5 μg of each DNA sample was used for each genomic DNA sample in Bisulfite Mix, DNA Protect Buffer and RNase-free water. A thermal cycler was then used to perform the bisulfite DNA conversion according to the following program: 99 °C 5 min, 60 °C 25 min, 99 °C 5 min, 60 °C 85 min, 99 °C 5 min, 60 °C 175 min, and 20 °C hold. 

To examine the methylation status at CpG islands of DLEC1, bisulfite sequencing was carried out. The bisulfite-conversed DNA was amplified for DLEC1 promoter with primers DLEC1BGS1/8 (Table 1). The primers gave a fragment of 291 bp, including 19 CpG sites. The temperature profiles for PCR amplification were as follows: 95 °C 5 min, 35 cycles of denaturing at 95 °C for 30 s, annealing at 50 °C for 45 s, extension at 72 °C for 30 s, and a final extension at 72 °C for 10 min. The amplified fragments were purified with QIAquick PCR Purification Kit (QIAGEN) and cloned with pGEM(R)-T Easy Vector System I (Promega). 6–8 clones of each sample were sequenced for identifying the original methylation status of the contained CpG sites. 

### 3.9. Chromatin Immunoprecipitation (ChIP)

ChIP assay was performed with the EZ-ChIP kit (#17-371, Upstate, Millipore), according to the instruction manual. Briefly, the adherent cells after different treatments, or transfected with different plasmids, were first crosslinked by adding formaldehyde directly into culture medium to a final concentration of 1% followed by incubation at room temperature for 10 min. After that, 1 mL of 10 × Glycine was added to quench unreacted formaldehyde. Cells were then washed twice with ice-cold 1 × PBS containing protease inhibitors and scraped, collected and pelleted at 4 °C 700 g for 2–5 min. Cell pellets were resuspended in 1ml of SDS lysis buffer with protease inhibitors and aliquoted 300–400 μL/tube, followed by sonication to shear DNA. The optimal conditions required for shearing crosslinked DNA to 200–1000 bp in length need to be determined for different cell lines. After sonication, samples were centrifuged at a minimum of 10000 g but not exceeding 15000 g at 4 °C for 10 min to remove insoluble materials and then aliquoted to 1 × 10^6^ cells of lysate for one immunoprecipitation. Each aliquot was diluted to 1 ml with dilution buffer containing protease inhibitors, and precleared by adding 60 μL of Protein G Agarose 50% slurry by incubating for 1 h at 4 °C with rotation. Agarose was pelleted by brief centrifugation (3000–5000 g for 1 min), and 1 mL supernatant was collected into fresh tube, with 10 μL (1%) of the supernatant removed as input control and saved at 4 °C. Immunoprecipitation antibodies ChIP Ab Acetyl-Histone H4 (#17-630, Millipore) and ChIP Ab Acetyl-Histone H3 (#17-615, Millipore) were added to the 1 ml supernatant and incubated overnight at 4 °C with rotation. After that, another 60 μL of Protein G Agarose was added to each IP and incubated for 1 h at 4 °C with rotation, so as to collect the antibody/histone complex. The protein G agarose/antibody/chromatin complex was pelleted by brief centrifugation and washed by a series of cold washing buffers. The immunocomplexes were then eluted with buffer, and DNA was released from protein/DNA complexes by reverse crosslink. The immunoprecipitated DNA was purified using spin columns and analyzed by PCR with primers DLEC1ChIPf/r (Table 1).

## 4. Conclusions

In conclusion, we have established a cell system to investigate the effects of epigenetic modifications on expression of target genes, in particular DLEC1, which is silenced in human cancers including HCC. This system was then expanded to reveal the effect of HBx on the DLEC1 expression in a genotype specific manner. HBx genotype A was found to decrease the DNA methylation, which in turn led to increased DLEC1 expression. Our results provide new insights on the impact of HBV genotypes on the epigenetic modulation of HBV-mediated hepatocarcinogenesis.
